# Exogenous fatty acids affect membrane properties and cold adaptation of *Listeria monocytogenes*

**DOI:** 10.1038/s41598-022-05548-6

**Published:** 2022-01-27

**Authors:** Alexander Flegler, Janice Iswara, Anna Tatjana Mänz, Frieda Sophia Schocke, Wanda Antonia Faßbender, Georg Hölzl, André Lipski

**Affiliations:** 1 Department of Food Microbiology and Hygiene, Institute of Nutritional and Food Science (IEL), University of Bonn, 53115 Bonn, Germany; 2 Department of Molecular Biotechnology, Institute of Molecular Physiology and Biotechnology of Plants (IMBIO), University of Bonn, 53115 Bonn, Germany

**Keywords:** Food microbiology, Bacteriology, Pathogens

## Abstract

*Listeria monocytogenes* is a food-borne pathogen that can grow at very low temperatures close to the freezing point of food and other matrices. Maintaining cytoplasmic membrane fluidity by changing its lipid composition is indispensable for growth at low temperatures. Its dominant adaptation is to shorten the fatty acid chain length and, in some strains, increase in addition the menaquinone content. To date, incorporation of exogenous fatty acid was not reported for *Listeria monocytogenes*. In this study, the membrane fluidity grown under low-temperature conditions was affected by exogenous fatty acids incorporated into the membrane phospholipids of the bacterium. *Listeria monocytogenes* incorporated exogenous fatty acids due to their availability irrespective of their melting points. Incorporation was demonstrated by supplementation of the growth medium with polysorbate 60, polysorbate 80, and food lipid extracts, resulting in a corresponding modification of the membrane fatty acid profile. Incorporated exogenous fatty acids had a clear impact on the fitness of the *Listeria monocytogenes* strains, which was demonstrated by analyses of the membrane fluidity, resistance to freeze-thaw stress, and growth rates. The fatty acid content of the growth medium or the food matrix affects the membrane fluidity and thus proliferation and persistence of *Listeria monocytogenes* in food under low-temperature conditions.

## Introduction

*Listeria monocytogenes* (*L*. *monocytogenes*) is responsible for the food-borne illness listeriosis, which causes a high proportion of severe cases and deaths worldwide^1–3^. Efforts to control this pathogen, which has a high mortality rate, are thwarted because *L*. *monocytogenes* is characterized by high tolerance to various environmental factors such as extreme temperatures^3–7^. The best-known and investigated characteristic of *L*. *monocytogenes* is its ability to grow in an extensive temperature range of − 1.5–50 °C^8–11^. This capacity of *L*. *monocytogenes* seems crucial for surviving in the natural environment for long periods, associated with colonization, reproduction, and persistence in the food-processing environment and on food-processing equipment^12^. Therefore, recent and recurring outbreaks revealed the importance of risk assessment analyses, which must also include the impact of the food matrix on growth rates and the low-temperature resilience of this organism^12–15^.

Exogenous fatty acid metabolism of various food pathogens and other bacterial species has been frequently studied in the context of pathogenicity or colonization of food matrices^16–22^. However, for temperature adaptation, the modification of the fatty acid profile in *L*. *monocytogenes* is achieved only by de novo synthesis of the branched-chain fatty acids and not by modifying the existing acyl chains^9,10,23,24^. Although exogenous fatty acids have already been detected in the fatty acid profile of *L*. *monocytogenes*^25^, there is no experimental evidence for incorporation in the membrane and the adaptive effect of exogenous fatty acids. Even homologous genes for the uptake and incorporation of exogenous fatty acids are present in *L*. *monocytogenes*, as in *Staphylococcus aureus* (*S*. *aureus*)^17^*.* Incorporating external fatty acids with a low melting point would represent an attractive explanation for the remarkable adaptation of the organism to low temperatures and the successful colonization of refrigerated food.

Here, we uncovered an unknown adaptation mechanism in *L*. *monocytogenes* by adding exogenous fatty acids. *L*. *monocytogenes* can non-selectively utilize and incorporate exogenous fatty acids into the polar lipids of the cell membrane from the environment in addition to de novo synthesis of fatty acids. The utilization has a negative or positive effect on the adaptation of the membrane to low growth temperatures. Correct acyl chain composition of the membrane is crucial for the survival of *L*. *monocytogenes* in the environment, and modification of this composition by exogenous fatty acids affects the fitness of this bacterium. This observation reveals a so far unconsidered impact of the lipid composition of the growth matrix on the growth and robustness of *L*. *monocytogenes* under low-temperature conditions. The potentially beneficial effect of food lipids on *L*. *monocytogenes* membranes may explain the successful colonization of many fatty food matrices stored under low-temperature conditions.

## Results

### *Listeria monocytogenes* covalently incorporates exogenous fatty acids into its membrane

Food matrices usually contain fatty acids ester-linked to triglycerides or polar lipids such as phospholipids or glycolipids. Therefore, in this study, we used polysorbate 60 (P60) and polysorbate 80 (P80) as well as lipid extracts from milk (ME), minced meat (MME), and smoked salmon (SSE) as supplements. Before the cultivation experiments, we analyzed fatty acid composition for tryptic soy broth-yeast extract medium (TSB-YE) without and with supplementation. P60 was used as a source for octadecanoic acid (C_18:0_) and P80 as a source for *cis*-9-octadecenoic acid (C_18:1_ *cis* 9). Both fatty acids could not be synthesized by *L*. *monocytogenes* and represent lipids with a high and a low melting temperature (*T*_*m*_), 69.3 °C for C_18:0_ and 12.8 °C for C_18:1_ *cis* 9. We used supplementation with d-sorbitol to control for the effects of the sorbitan moiety of the polysorbate additions. The results for d-sorbitol controls coincide with those from cultures without any supplement. d-sorbitol was considered not to affect fatty acid profiles, membrane fluidity, or cell fitness. In addition, supplementation did not affect the medium's water activity (*a*_*w*_) and pH. We cultivated three *L*. *monocytogenes* strains at 6 and 37 °C in TSB-YE without or with supplementation and analyzed the impact on the bacterial fatty acids composition.

The dominant fatty acids of the TSB-YE supplemented with P60 were 54.4 ± 0.9% C_18:0_ and 46.6 ± 0.6% hexadecanoic acid (C_16:0_), TSB-YE supplemented with P80 consisted of 75.3 ± 0.5% C_18:1_ *cis* 9, 18.7 ± 0.3% C_16:0_ and 6.0 ± 0.3 *cis*-9-hexadecenoic acid (C_16:1_ *cis* 9), and TSB-YE supplemented with P60P80 were 49.0 ± 0.7% C_18:1_ *cis* 9, 29.2 ± 0.2% C_16:0_, and 21.8 ± 1.0% C_18:0_, respectively. We could detect no fatty acids in the TSB-YE without supplementation and with d-sorbitol supplementation. After growth in the previously analyzed growth media, all *L*. *monocytogenes* strains showed a branched-chain fatty acid profile at 6 and 37 °C growth temperature (Tables 1, 2).

**Table 1:** Fatty acid (FA) composition, weighted-average melting temperature (WAMT), the ratio of *anteiso*-C_15:0_ to *anteiso*-C_17:0_ (Ri_ai-15/ai-17_), and menaquinone-7 (MK-7) content.

Parameter	DSM 20600^T^				FFH				FFL 1			
	TSB-YE	P60	P60P80	P80	TSB-YE	P60	P60P80	P80	TSB-YE	P60	P60P80	P80
**FA (%)**												
*anteiso*-C_13:0_	n.d	6.9 ± 0.9**	n.d	n.d	n.d	0.2 ± 0.2	n.d	n.d	n.d	0.7 ± 0.1	n.d	n.d
*iso*-C_14:0_	0.8 ± 0.3	0.4 ± 0.3	n.d	0.2 ± 0.2	n.d	0.7 ± 0.2	0.2 ± 0.2	n.d	1.2 ± 0.2	1.3 ± 0.2	0.9 ± 0.2	1.3 ± 1.3
C_14:0_	n.d	0.5 ± 0.4	n.d	n.d	n.d	0.3 ± 0.1	n.d	n.d	0.4 ± 0.3	0.3 ± 0.1	0.2 ± 0.0	0.1 ± 0.1
*iso*-C_15:0_	8.8 ± 1.7	1.5 ± 0.4***	3.5 ± 0.6*	4.9 ± 1.0	9.7 ± 1.7	4.3 ± 0.5***	3.4 ± 0.6****	2.9 ± 0.5****	5.3 ± 0.2	5.0 ± 0.5	4.7 ± 0.7	6.9 ± 3.2
*anteiso*-C_15:0_	82.6 ± 2.8	30.9 ± 3.6****	41.4 ± 5.4****	67.1 ± 8.9****	85.1 ± 0.6	39.6 ± 5.3****	37.9 ± 2.9****	68.8 ± 3.5****	88.5 ± 0.5	36.8 ± 1.6****	45.5 ± 1.7****	66.1 ± 10.3****
*iso*-C_16:0_	0.7 ± 0.2	n.d	n.d	0.2 ± 0.2	0.3 ± 0.3	1.1 ± 0.1	0.6 ± 0.1	0.1 ± 0.1	1.1 ± 0.9	1.5 ± 0.4	1.2 ± 0.2	0.8 ± 0.7
C_16:0_	n.d	42.0 ± 1.6****	14.3 ± 3.6****	2.5 ± 0.9	n.d	21.3 ± 0.8****	17.4 ± 1.6****	1.9 ± 0.8	0.2 ± 0.2	22.7 ± 0.5****	12.1 ± 1.4****	2.9 ± 2.6
*anteiso*-C_17:0_	7.1 ± 0.8	1.8 ± 0.4**	7.5 ± 1.0	8.0 ± 1.7	4.9 ± 1.8	11.3 ± 1.5****	8.0 ± 0.6	9.6 ± 0.6**	3.4 ± 1.4	9.5 ± 0.7***	10.5 ± 0.7****	8.6 ± 0.2**
C_18:1_ *cis* 9	n.d	n.d	14.8 ± 2.4****	17.1 ± 5.3****	n.d	n.d	14.4 ± 0.9****	16.7 ± 3.1****	n.d	n.d	15.5 ± 0.7****	12.8 ± 3.0****
C_18:0_	n.d	16.7 ± 4.9****	18.6 ± 5.6****	n.d	n.d	21.0 ± 5.3****	18.0 ± 3.7****	n.d	n.d	22.3 ± 2.9****	9.5 ± 2.6****	n.d
**WAMT (°C)**	27.9 ± 0.7	47.2 ± 2.6****	38.2 ± 3.9****	25.6 ± 0.4	27.4 ± 0.1	45.0 ± 2.3****	39.5 ± 2.1****	25.0 ± 0.2	26.9 ± 0.3	46.3 ± 1.1****	34.6 ± 1.4****	27.3 ± 2.3
**Ri**_**ai-15/ai-17**_	11.6	16.7	5.6	7.5	17.3	3.5	4.7	7.2	26.4	3.9	4.3	7.7
**MK-7 (nmol g**^**-1**^**)**	213 ± 12	213 ± 7	188 ± 19*	175 ± 16***	181 ± 9	178 ± 7	170 ± 4	135 ± 5****	89 ± 5	139 ± 2****	135 ± 8****	90 ± 8

*Listeria monocytogenes* strains DSM 20600^T^, FFH, and FFL 1 grown at 6 °C in tryptic soy broth-yeast extract medium without supplementation (TSB-YE), with 0.1% (wt/vol) polysorbate 60 (P60), with 0.05% (wt/vol) each of polysorbate 60 and polysorbate 80 (P60 P80), and with 0.1% (wt/vol) polysorbate 80 (P80). Values are means ± standard deviation (*n* = 3). Asterisks represent *p* values (*p < 0.001, **p < 0.0001, ***p < 0.00001, ****p < 0.000001) compared to cultures in TSB-YE without supplementation.

**Table 2:** Fatty acid (FA) composition, weighted-average melting temperature (WAMT), and the ratio of *anteiso*-C_15:0_ to *anteiso*-C_17:0_ (Ri_ai-15/ai-17_).

Parameter	DSM 20600^T^				FFH				FFL 1			
	TSB-YE	P60	P60P80	P80	TSB-YE	P60	P60P80	P80	TSB-YE	P60	P60P80	P80
**FA (%)**												
*iso*-C_14_:0	n.d	0.4 ± 0.1	n.d	n.d	0.2 ± 0.0	0.4 ± 0.1	n.d	n.d	0.2 ± 0.0	0.5 ± 0.1	n.d	n.d
C_14:0_	n.d	n.d	n.d	n.d	n.d	0.4 ± 0.4	n.d	n.d	n.d	0.2 ± 0.2	n.d	n.d
*iso*-C_15:0_	3.9 ± 0.1	6.0 ± 0.2****	5.8 ± 0.1***	5.8 ± 0.0***	9.3 ± 0.6	6.5 ± 0.2****	6.5 ± 0.1****	6.5 ± 0.0****	6.5 ± 0.2	5.7 ± 0.3	6.6 ± 0.0	6.3 ± 0.1
*anteiso*-C_15:0_	60.2 ± 1.1	40.6 ± 0.3****	39.7 ± 0.4****	40.0 ± 0.6****	52.5 ± 1.9	38.0 ± 1.1****	38.7 ± 0.2****	37.8 ± 0.4****	49.6 ± 1.6	38.6 ± 0.7****	39.8 ± 0.2****	39.4 ± 0.4****
*iso*-C_16:0_	1.7 ± 0.1	2.7 ± 0.1	2.6 ± 0.0	2.6 ± 0.1	2.5 ± 0.3	3.0 ± 0.1	2.8 ± 0.2	3.3 ± 0.1	3.6 ± 0.2	3.6 ± 0.2	3.4 ± 0.1	3.8 ± 0.1
C_16:0_	0.6 ± 0.1	6.7 ± 0.3****	3.8 ± 0.1****	2.7 ± 0.2****	1.3 ± 0.3	10.5 ± 0.5****	5.7 ± 0.2****	4.1 ± 0.0****	1.3 ± 0.1	7.6 ± 1.5****	4.6 ± 0.2****	3.6 ± 0.3****
*iso*-C_17:0_	1.3 ± 0.1	3.1 ± 0.0***	3.5 ± 0.1****	3.4 ± 0.1****	2.4 ± 0.3	3.6 ± 0.1**	4.0 ± 0.1***	4.2 ± 0.1****	1.4 ± 0.2	3.2 ± 0.2**	3.4 ± 0.0***	3.3 ± 0.2***
*anteiso*-C_17:0_	32.3 ± 0.9	39.0 ± 0.1****	41.2 ± 0.2****	42.2 ± 0.7****	31.9 ± 0.7	36.1 ± 1.0****	38.7 ± 0.4****	40.4 ± 0.2****	37.5 ± 0.9	36.1 ± 0.7*	39.4 ± 0.0***	40.2 ± 0.2****
C_18:1_ cis 9	n.d	n.d	2.5 ± 0.0****	3.4 ± 0.2****	n.d	n.d	3.1 ± 0.3****	3.6 ± 0.1****	n.d	n.d	2.5 ± 0.2****	3.3 ± 0.3****
C_18:0_	n.d	1.5 ± 0.3**	0.8 ± 0.2	n.d	n.d	1.5 ± 0.3	0.5 ± 0.1***	n.d	n.d	4.5 ± 2.1****	0.4 ± 0.0	n.d
**WAMT (°C)**	30.2 ± 0.5	34.4 ± 0.1****	32.7 ± 0.2****	31.9 ± 0.0****	32.1 ± 0.6	35.7 ± 0.3****	33.0 ± 0.2*	32.5 ± 0.0	32.5 ± 0.4	36.1 ± 0.6****	33.1 ± 0.1	32.7 ± 0.1
**Ri**_**ai-15/ai-17**_	1.9	1.0	1.0	0.9	1.6	1.1	1.0	0.9	1.3	1.1	1.0	1.0

*Listeria monocytogenes* strains DSM 20600^T^, FFH, and FFL 1 grown at 37 °C in tryptic soy broth-yeast extract medium without supplementation (TSB-YE), with 0.1% (wt/vol) polysorbate 60 (P60), with 0.05% (wt/vol) each of polysorbate 60 and polysorbate 80 (P60P80), and with 0.1% (wt/vol) polysorbate 80 (P80). Values are means ± standard deviation (*n* = 3). Asterisks represent *p* values (*p < 0.001, **p < 0.0001, ***p < 0.00001, ****p < 0.000001) compared to cultures in TSB-YE without supplementation.

The dominating fatty acids of all strains were 12 methyltetradecanoic acid (*anteiso*-C_15:0_), 14-methylhexadecanoic acid (*anteiso*-C_17:0_), and 13-methyltetradecanoic acid (*iso*-C_15:0_), at both growth temperatures. The three branched-chain fatty acids accounted for at least 96% of the total fatty acids in all strains when grown in TSB-YE without supplementation at 6 or 37 °C, respectively (Tables 1, 2). All strains significantly reduced the three branched-chain fatty acids after cultivation at 6 and 37 °C in TSB-YE supplemented with P60, with P80, or with P60P80. Additionally, the fatty acid profile presents the exogenous fatty acids C_16:0_, C_18:0_, and C_18:1_ *cis* 9. For all strains, the content of C_16:0_ and C_18:0_ was nearly 42–59% (6 °C), and 8–12% (37 °C) after supplementation with P60, of C_16:0_ and C_18:1_ *cis* 9 was nearly 16–20% (6 °C), and 6–8% (37 °C) after supplementation with P80, and of C_16:0_, C_18:0_ and C_18:1_ *cis* 9 was nearly 37–50% (6 °C) and 7–9% (37 °C) after supplementation with P60P80. Calculation of the weighted average melting temperature (WAMT) based on all detected fatty acids for each profile demonstrated that the supplemented fatty acids affected the membrane melting temperature. During the growth of the tested strains, the proportion of exogenous fatty acids in cell extracts increased with an increasing optical density at 625 nm (OD_625_) at both incubation temperatures indicating the accumulation of exogenous fatty acids in the bacterial membrane (data not shown).

The three dominant fatty acids of the TSB-YE supplemented with milk extract (ME) were 25.6 ± 0.5% C_16:0_, 18.8 ± 0.2% C_18:1_ *cis* 9, and 15.0 ± 0.2% tetradecanoic acid (C_14:0_), of TSB-YE supplemented with minced meat extract (MME) were 36.7 ± 5.0% C_18:1_ *cis* 9, 28.3 ± 0.2% C_16:0_, and 11.8 ± 2.1% C_18:0_, and of TSB-YE supplemented with smoked salmon extract (SSE) were 16.2 ± 1.7% *cis*-9,12-octadecadienoic acid (C_18:2_ *cis* 9,12), 15.5 ± 4.0% C_18:1_ *cis* 9, and 14.3 ± 1.2% C_16:0_ (Table 2). After growth in the presence of lipid extracts from food, fatty acid profiles of all *L*. *monocytogenes* strains contained exogenous fatty acids from the supplemented food extracts. After growth in TSB-YE supplemented with ME, with MME, or with SSE, all strains showed a reduction of branched-chain fatty acids and the presence of exogenous fatty acids. In all three strains, the exogenous fatty acids dodecanoic acid (C_12:0_), C_14:0_, C_16:1_ *cis* 9, C_16:0_, C_18:2_ *cis* 9,12, C_18:1_ *cis* 9, *cis*-11-octadecadienoic acid (C_18:1_ *cis* 11), C_18:0_, *cis*-5,8,11,14,17-eicosapentaenoic acid (C_20:5_)_,_ and *cis*-4,7,10,13,16,19-docosahexaenoic acid (C_22:6_) of the food lipid extracts were detected (Table 2). The content of exogenous fatty acids in the total fatty acid profile was about 10–22% after supplementation with ME, about 10–25% after supplementation with MME, and about 12–28% after supplementation with SSE for all strains at 6 °C growth temperature.

Because the cells' menaquinone-7 (MK-7) content was previously associated with membrane fluidity under low-temperature conditions, we analyzed this lipid for all cultures grown at 6 °C (Table 1). *L*. *monocytogenes* strain DSM 20600^T^, FFH, and FFL 1 had an MK-7 content of 213 ± 12, 181 ± 9, and 89 ± 5 nmol g^−1^, respectively, after growth in TSB-YE at 6 °C (Table 1). This MK-7 content is in accord with previous findings that the former two strains increase their production under low-temperature growth conditions, but the latter strain does not^26^. After growth in TSB-YE supplemented with P80, strains DSM 20600^T^ and FFH had significantly lower MK-7 content of 175 ± 16 and 135 ± 5 nmol g^−1^, respectively, while strain FFL 1 showed unchanged MK-7 content of 90 ± 8 nmol g^−1^. After growth in TSB-YE supplemented with P60 or P60P80, strain FFL 1 was the only strain that significantly increased MK-7 content of 139 ± 2 and 135 ± 8 nmol g^−1^, respectively. For the other two strains, supplementation with P60 or P60P80 did not increase the MK-7 content. Thus, supplementation with exogenous fatty acids also affects the fatty acid synthesis of *L*. *monocytogenes*. We used the ratio of *anteiso*-C_15:0_ to *anteiso*-C_17:0_ (Ri_ai-15/ai-17_) to assess the impact of exogenous fatty acids on the profile of endogenously synthesized fatty acids. For all supplemented cultures, except one, we noticed the decrease of the Ri_ai-15/ai-17_. Strain *L. monocytogenes* DSM 20600^T^ grown at 6 °C and supplemented with P60 was the only exception. Thus, the WAMT of the total fatty acid profiles was not primarily determined by the shift of the Ri_ai-15/ai-17_ but by the *T*_*m*_ of the incorporated exogenous fatty acids (Tables 1, 2, 3).

**Table 3:** Fatty acid (FA) composition, weighted-average melting temperature (WAMT), and the ratio of *anteiso*-C_15:0_ to *anteiso*-C_17:0_ (Ri_ai-15/ai-17_).

Parameter	Food lipid			DSM 20600^T^			FFH			FFL 1		
	ME	MME	SSE	ME	MME	SSE	ME	MME	SSE	ME	MME	SSE
**FA (%)**												
C_12:0_	7.0 ± 0.4			0.7 ± 0.0			0.4 ± 0.1			0.9 ± 0.2		
C_14:0_	15.0 ± 0.2	3.5 ± 0.6	7.0 ± 0.8	2.1 ± 0.4	0.7 ± 0.1	1.0 ± 0.2	2.6 ± 0.4	0.8 ± 0.3	0.9 ± 0.2	3.7 ± 1.1	0.7 ± 0.0	1.0 ± 0.2
*iso*-C_15:0_				8.7 ± 0.6	10.9 ± 0.1	10.3 ± 2.1	6.6 ± 0.2	5.8 ± 0.6	5.8 ± 1.6	5.8 ± 0.3	5.8 ± 0.6	5.6 ± 1.7
*anteiso*-C_15:0_				71.0 ± 0.4	66.6 ± 1.1	66.1 ± 0.9	71.1 ± 0.7	66.3 ± 0.2	57.8 ± 10.0	60.8 ± 7.9	56.8 ± 1.1	55.9 ± 9.8
C_16:1_ *cis* 9	2.0 ± 0.0	5.2 ± 0.9	6.2 ± 0.6	1.0 ± 0.1	0.3 ± 0.0	0.3 ± 0.1	0.7 ± 0.0	0.4 ± 0.0	1.6 ± 1.5	1.0 ± 0.0	1.2 ± 0.2	1.6 ± 1.3
*iso*-C_16:0_				0.3 ± 0.1	1.7 ± 0.2	1.2 ± 0.1	0.3 ± 0.2	0.8 ± 0.1	0.8 ± 0.2	0.5 ± 0.1	0.7 ± 0.0	1.1 ± 0.4
C_16:0_	25.6 ± 0.5	28.3 ± 0.2	14.3 ± 1.2	2.7 ± 0.0	2.2 ± 0.1	5.0 ± 2.1	3.2 ± 0.6	3.9 ± 0.5	10.1 ± 5.6	7.8 ± 3.2	6.1 ± 0.0	10.1 ± 5.2
*iso*-C_17:0_				0.4 ± 0.0	0.4 ± 0.0	0.4 ± 0.1	0.4 ± 0.1	0.4 ± 0.0	0.3 ± 0.1	0.4 ± 0.1	0.3 ± 0.0	0.3 ± 0.1
*anteiso*-C_17:0_				8.6 ± 0.4	10.7 ± 1.1	10.0 ± 0.4	10.2 ± 0.5	11.3 ± 0.3	9.9 ± 3.0	9.2 ± 0.3	11.3 ± 0.3	11.0 ± 1.4
C_18:2_ *cis* 9,12	3.0 ± 0.1	9.6 ± 1.7	16.2 ± 1.7	0.3 ± 0.1	1.5 ± 0.0	0.7 ± 0.1	0.3 ± 0.0	1.8 ± 0.0	3.6 ± 3.5	0.6 ± 0.1	2.7 ± 0.0	3.7 ± 2.5
C_18:1_ *cis* 9	18.4 ± 0.2	36.7 ± 5.0	15.5 ± 4.0	2.1 ± 0.2	2.3 ± 0.0	1.1 ± 0.3	2.0 ± 0.2	4.6 ± 0.0	5.3 ± 4.9	5.2 ± 1.8	9.7 ± 0.0	5.6 ± 3.5
C_18:1_ *cis* 11	1.3 ± 0.0	4.8 ± 0.8	2.5 ± 0.4		0.2 ± 0.0	0.2 ± 0.1		0.2 ± 0.0	1.6 ± 1.2		0.7 ± 0.0	1.2 ± 1.1
C_18:0_	10.0 ± 0.2	11.8 ± 2.1	2.8 ± 0.3	0.7 ± 0.5	0.8 ± 0.0	1.3 ± 1.0	0.9 ± 0.5	2.6 ± 0.0	1.2 ± 0.7	2.7 ± 1.7	3.1 ± 0.0	1.0 ± 0.5
C_20:5_			6.2 ± 1.4			0.3 ± 0.0			0.5 ± 0.3			0.5 ± 0.4
C_22:6_			10.5 ± 0.4			0.9 ± 0.3			0.8 ± 0.5			1.3 ± 0.6
**WAMT (°C)**				29.0 ± 0.8	29.4 ± 1.0	30.0 ± 3.5	29.1 ± 1.6	29.0 ± 1.0	28.9 ± 8.8	31.3 ± 6.4	28.9 ± 0.7	28.6 ± 7.6
**Ri**_**ai-15/ai-17**_				8.3	6.2	6.6	7.0	5.9	5.8	6.6	5.0	5.1

Tryptic soy broth-yeast extract medium supplemented with food lipid extracts and *Listeria monocytogenes* strains DSM 20600^T^, FFH, and FFL 1 grown at 6 °C on tryptic soy agar-yeast extract medium supplemented with 0.1% (wt/vol) milk extract (ME), with 0.1% (wt/vol) minced meat extract (MME) or with 0.1% (wt/vol) smoked salmon extract (SSE). Values are means ± standard deviation (*n* = 3).

**Figure 1:**
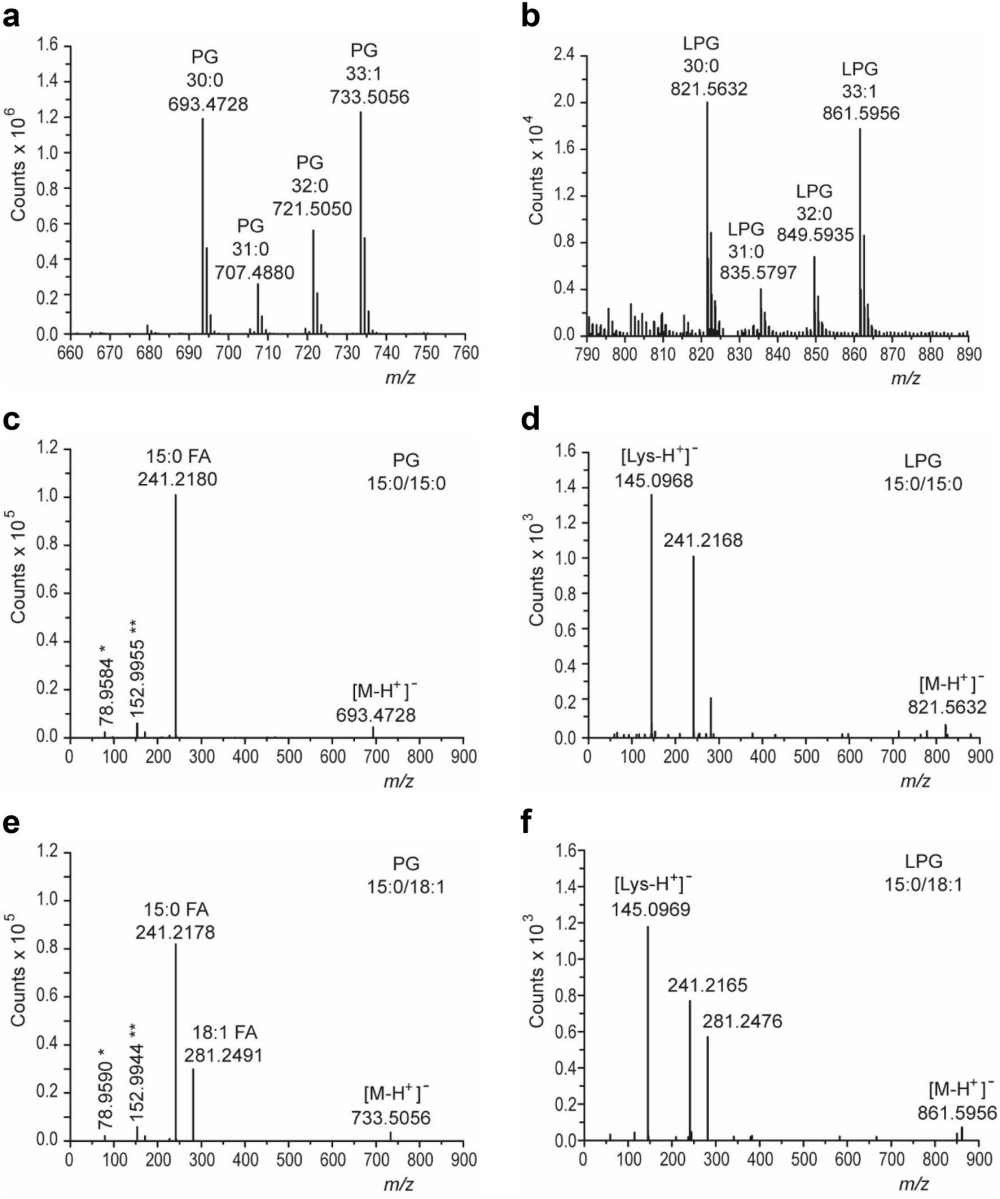
Analysis of polar lipids by quadrupole time-of-flight mass spectrometry. Phosphatidylglycerol (PG) and lysyl-phosphatidylglycerol (LPG) were detected in lipid extracts from cells of *Listeria monocytogenes* strain DSM 20600^T^ cells grown at 6 °C on tryptic soy agar-yeast extract medium supplemented with 0.1% (wt/vol) polysorbate 80. The lipids were measured in the negative ion mode. Different molecular species of PG (**a**) and LPG (**b**) can be observed in the total ion spectra. Characteristic fragments in the MS/MS spectra of the respective lipids allow the detection of single fatty acids as indicated in the figure (**c**–**f**). Further fragments are characteristic for PG derived from the phosphite anion *[PO_3_]¯ and the glycerolphosphate **[GroP]¯ head group. Fragmentation of LPG results in detecting a fragment (*m/z* 145.09) derived from a deprotonated lysyl residue [Lys-H^+^]¯^51,52^.

We could confirm the active incorporation of exogenous fatty acids in the bacterial cell membrane of *L*. *monocytogenes*. Polar lipids were extracted from all strains grown at 6 °C in TSB-YE supplemented with P80 and analyzed with quadrupole time-of-flight mass spectrometry (Q-TOF MS). Phosphatidylglycerol (PG)-C_30:0_ (*m/z* 693.47) and lysyl-phosphatidylglycerol (LPG)-C_30:0_ (*m/z* 821.56) were identified in all strains as main molecular species and contained only *iso*/*anteiso*-C_15:0_ fatty acids (*m/z* 241.21) as derived from the total and the MS/MS spectra (Fig. 1a–d). Two other main species, PG-C_33:1_ (*m/z* 733.50) and LPG-C_33:1_ (*m/z* 861.59), were detected (Fig. 1a,b), which were absent in strains grown without supplementation. The fragmentation patterns in the MS/MS spectra of the two lipids confirm the presence of C_15:0_ and, in addition, C_18:1_ as derived from the fragments with *m/z* 241.21 and *m/z* 281.24, respectively (Fig. 1e,f). These results confirm the incorporation of the exogenous fatty acid C_18:1_ into phospholipids in *L*. *monocytogenes*. We could not observe the incorporation of two C_18:1_ per lipid molecule. Other phospholipids' fatty acids were C_17:0_ (native) and C_16:0_ (supplemented). C_16:0_, C_17:0,_ and C_18:1_ were observed only in combination with C_15:0_ in the different lipid species but not among each other. Incorporation of C_18:0_ into PG and LPG was also observed. C_16:0_, C_18:0,_ and C_18:1_ could not be detected in strains grown without supplementation. C_18:1_ was not detectable in the two glycolipids, monoglycosyldiacylglycerol and diglycosyldiacylglycerol, found in the tested strains (data not shown), indicating a preference for incorporation of exogenous fatty acids into phospholipids.

### The incorporation of exogenous fatty acids alters membrane fluidity and support cold adaptation

**Figure 2:**
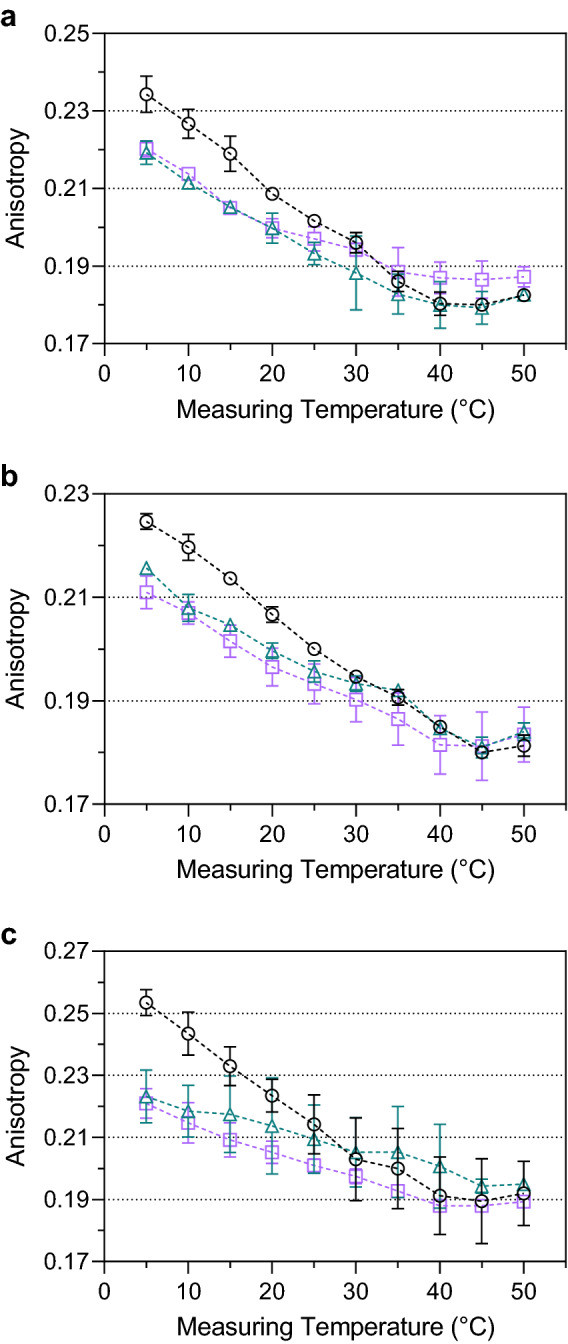
Analysis of membrane fluidity by TMA-DPH anisotropy. *Listeria monocytogenes* strains DSM 20600^T^ (**a**), FFH (**b**), and FFL 1 (**c**) grown at 6 °C in tryptic soy broth-yeast extract medium without supplementation (black circles), with 0.1% (wt/vol) of polysorbate 80 (green triangles), and with 0.1% (wt/vol) salmon lipid extract (purple squares). Values are means ± standard deviation (*n* = 3).

**Figure 3:**
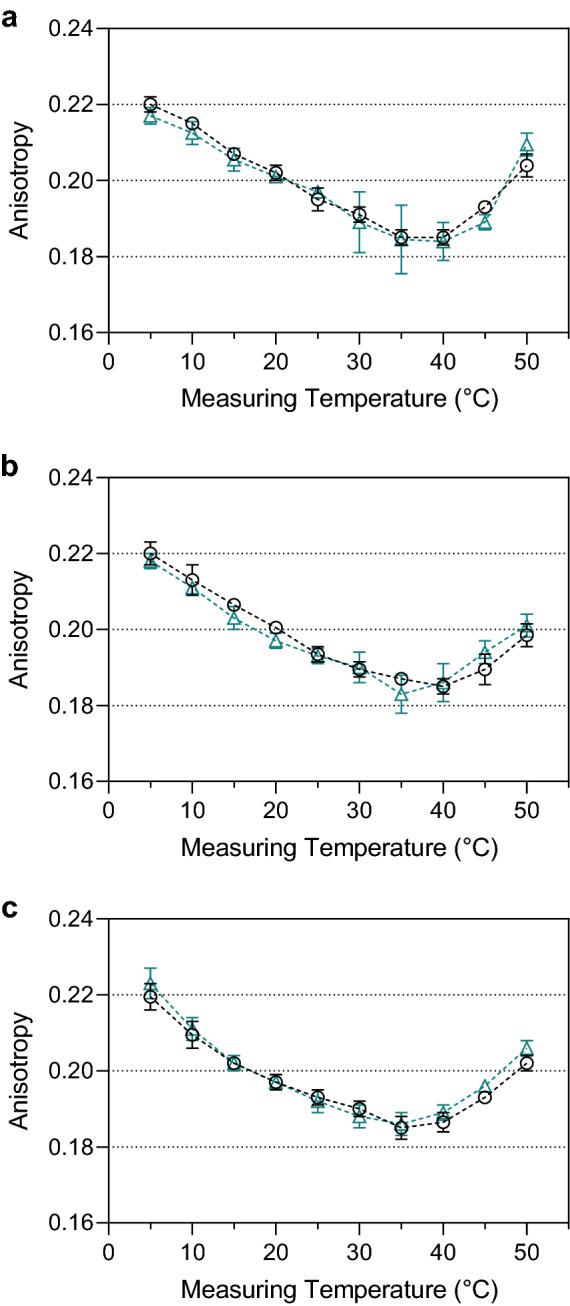
Time-dependent analysis of membrane fluidity by TMA-DPH anisotropy. *Listeria monocytogenes* strain DSM 20600^T^ incubated at 6 °C after 1 h (**a**), 24 h (**b**), and 48 h (**b**) in Ringer’s solution without supplementation (black circles) and with 0.1% (wt/vol) of polysorbate 80 (green triangles). Values are means ± standard deviation (*n* = 2).

We measured changes in the lateral diffusion capability of the cytoplasmic membranes induced by supplementation with P80 or SSE after growth at 6 °C based on trimethylammonium diphenylhexatriene (TMA-DPH) anisotropy (Fig. 2). The data showed an apparent discrepancy between cells grown with and without exogenous fatty acid supplementation. Supplementation with an exogenous fatty acid source during growth at 6 °C resulted in a higher fluidity of the membranes for all *L*. *monocytogenes* strains in a temperature range between 5 and 15 °C. The difference between supplemented and non-supplemented strains is > Δ0.010 at these low temperatures. At higher temperatures, membrane fluidity increased steadily until TMA-DPH anisotropy values for all three strains approximate each other at temperatures of 20 °C and above. *L*. *monocytogenes* strains grown at 6 °C in TSB-YE with P80 or SSE as an exogenous source of fatty acids showed a significantly smaller TMA-DPH anisotropy change over the entire measuring range, which indicates a broad transition phase. Strain FFL 1 showed the most considerable effect from all three strains. Growth with P80 and SSE increased cell membrane fluidity and a broader transition phase than strains without supplementation by exogenous fatty acids. This is because these supplements provide fatty acids with low *T*_*m*_. Long-term incubations for up to 48 h of non-growing cells suspended in Ringer's solution with P80 at 6 °C showed no effect on membrane fluidity (Fig. 3). These results indicated the dependency of membrane effects on growing, biochemically active cells but not on the mere abiotic association of P80 with the cell membrane. In addition, for this long-term incubation, C_18:1_ could not be detected in the cells' fatty acid profile, indicating that fatty acid profile analyses show only incorporated fatty acids but no fatty acids bound to polysorbate.

### Exogenous fatty acids affect resistance to freeze-thaw stress and growth rates

**Figure 4:**
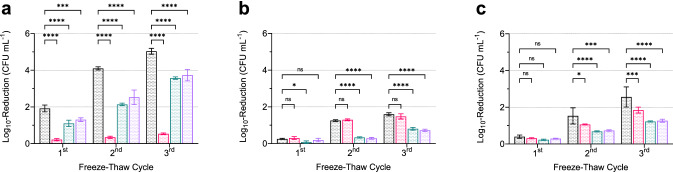
Logarithmic reduction of viable cell counts after freeze-thaw stress test. *Listeria monocytogenes* strains DSM 20600^T^ (**a**), FFH (**b**), and FFL 1 (**c**) grown at 6 °C in tryptic soy broth-yeast extract medium (TSB-YE) without supplementation (black), with 0.1% (wt/vol) polysorbate 60 (red), with 0.1% (wt/vol) polysorbate 80 (green), and with 0.1% (wt/vol) salmon lipid extract (indigo) after one, two and three freeze-thaw cycles (each 24 h) relative to the initial cell count. Values are means ± standard deviation (*n* = 3). Asterisks represent *p* values (*p < 0.001, **p < 0.0001, ***p < 0.00001, ****p < 0.000001) compared to cultures in TSB-YE without supplementation.

We applied a freeze-thaw stress test as an indicator of membrane integrity. This test showed a positive and a negative impact of exogenous fatty acids sources on cell resistance depending on the supplement (Fig. 4). After growth at 6 °C and subjected to freeze-thaw stress, all *L*. *monocytogenes* strains showed a significant decrease of log_10_-reduction of CFU mL^−1^ if supplemented with P60, P80, or SSE compared to non-supplemented TSB-YE. Strains DSM 20600^T^ and FFL 1 grown in TSB-YE with P60 and all strains grown in TSB-YE with P80 showed significantly decreased log_10_-reduction after freeze-thaw stress compared to cultures in un-supplemented TSB-YE. Thus, supplementation with exogenous fatty acids can positively affect cell fitness, regardless of the *T*_*m*_ of the incorporated fatty acids. The highest resistance against freeze-thaw stress was observed for strain DSM 20600^T^ after growth in TSB-YE with P60 and for strains FFH and FFL 1 after growth in TSB-YE with P80 or SSE. Supplementation with SSE produced the exact extent of log_10_-reduction of CFU mL^−1^ as supplementation with P80 for all strains.

**Figure 5:**
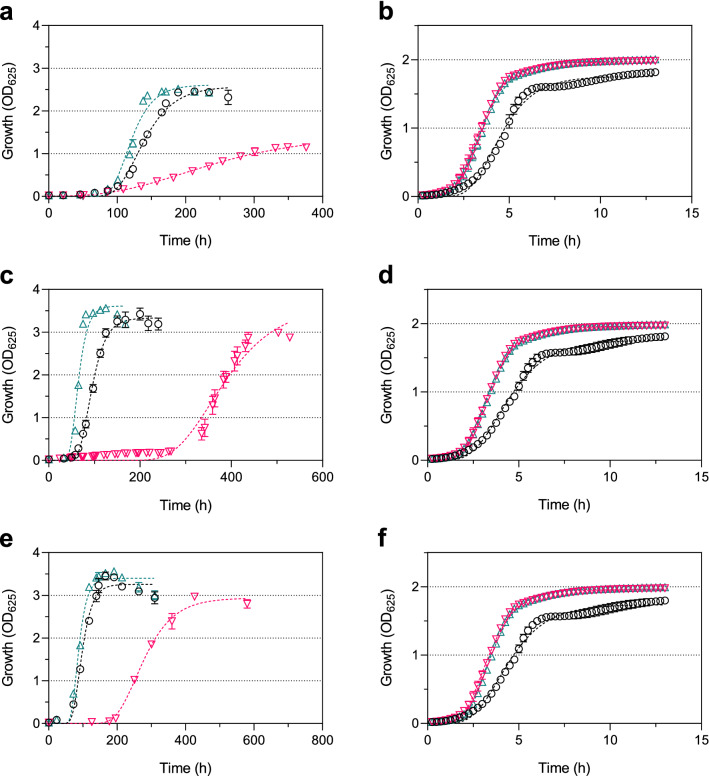
Growth kinetics. *Listeria monocytogenes* strains DSM 20600^T^ (**a**, **b**), FFH (**c**, **d**) and FFL 1 (**e**, **f**) grown at 6 °C (**a**, **c**, **e**) and 37 °C (**b**, **d**, **f**) in tryptic soy broth-yeast extract medium without supplementation (black circles), with 0.1% (wt/vol) of polysorbate 60 (red inverted triangles), and with 0.1% (wt/vol) of polysorbate 80 (green triangles) Values are means ± standard deviation (*n* = 3 at 6 °C; *n* = 8 at 37 °C).

The supplementation experiments showed a clear impact on growth rates of the tested *L*. *monocytogenes* strains at 6 and 37 °C (Fig. 5). The growth rates at 6 °C were reduced after supplementation with P60 and increased after supplementation with P80, compared to non-supplemented controls. The growth rates decreased from 0.034 to 0.010, 0.048 to 0.013, and 0.048 to 0.017 after supplementation with P60 and increased to 0.047, 0.072, and 0.061 after supplementation with P80 in strains DSM 20600^T^, FFH, and FFL 1 grown at 6 °C, respectively. In contrast to cultures grown at 6 °C, an increase of growth rates could be demonstrated for all strains at 37 °C after supplementation with P60 and with P80. Growth rates increased from 0.72 to 0.95 and 0.93 for strain DSM 20600^T^, from 0.61 to 0.97 and 0.94 for strain FFH, and from 0.63 to 0.96 and 1.0 for strain FFL 1, grown at 37 °C after supplementation with P60 or P80, respectively. Thus, the exogenous fatty acid with high *T*_*m*_ (C_18:0_) inhibits growth at 6 °C for all strains, whereas a fatty acid with low *T*_*m*_ (C_18:1_ *cis* 9) positively affected growth rates. In contrast, both types of fatty acids positively affected growth rates at 37 °C. These growth rates are in accord with our observation that all three strains showed faster colony formation at 6 °C growth temperature on tryptic soy agar-yeast extract medium (TSA-YE) if supplemented with ME, MME, or SSE, reflecting the positive influence of exogenous fatty acids from foods.

## Discussion

Some foods are known to have an increased risk for contamination with *L. monocytogenes* even under low-temperature storage conditions^1,12^. We used P60 with a high *T*_*m*_ fatty acid (C_18:0_, *T*_*m*_ 69.2 °C) and P80 with a low *T*_*m*_ fatty acid (C_18:1_ *cis* 9, *T*_*m*_ 12.8 °C) as supplements. Straight chain fatty acids, saturated and unsaturated, were not synthesized by *L*. *monocytogenes*. Therefore the incorporated C_18:0_ and C_18:1_ *cis* 9 must be exogenous in origin^27–30^. The same is true for other unsaturated fatty acids supplemented with the food extracts such as C_18:2_ *cis* 9,12 with a *T*_*m*_ of − 7.2 °C and C_18:1_ *cis* 11 with a *T*_*m*_ of 15.4 °C. The three *L*. *monocytogenes* strains studied showed a temperature-dependent change of their fatty acid profiles (Tables 1, 2) which were in accord with the previous reports^9,10,23,26,31^. The adaptation mechanism shifted the Ri_ai-15/ai-17_ from *anteiso*-C_17:0_ (*T*_*m*_ 37.1 °C) to *anteiso*-C_15:0_ (*T*_*m*_ 24.1 °C). *L*. *monocytogenes* strain DSM 20600^T^ and FFH showed a less pronounced fatty acid shift at lower growth temperatures than strain FFL 1 (Tables 1, 2). As described before, MK-7 is an additional modulator of membrane fluidity for these strains and is crucial for bacterial cell fitness^26,31^. However, all strains showed an expansion of their fatty acid profiles after supplementation with exogenous fatty acids as these were assimilated by all *L*. *monocytogenes* strains (Tables 1, 2, 3). All strains were even able to incorporate polyunsaturated fatty acids such as C_20:5_ with a *T*_*m*_ of − 53 °C and C_22:6_ with a *T*_*m*_ of − 44 °C derived from the supplemented SSE. The bactericidal effects of polyunsaturated fatty acids^32^, as previously described, did not occur in *L*. *monocytogenes*. As expected, exogenous fatty acids with lower *T*_*m*_ and those with higher *T*_*m*_ such as C_14:0_ with a *T*_*m*_ of 53.5 °C and C_16:0_ with a *T*_*m*_ of 62.2 °C were incorporated. We found no indication for selective incorporation of particular fatty acids. The supplementation with an equimolar mixture of P60 and P80 showed no favored incorporation of the lower melting point fatty acid in all strains at low-temperature growth conditions. All strains did not selectively incorporate the supplemented fatty acids according to their *T*_*m*_, but their percentage availability in the medium (Tables 1, 2, 3). This finding is also in accord with the increasing appearance of exogenous fatty acids in the fatty acid profiles of the strains during cultivation (data not shown).

Exogenous fatty acids replaced endogenously synthesized fatty acids and affected the fatty acid synthesis of *L. monocytogenes*. We found a decrease of the Ri_ai-15/ai-17_ in the presence of exogenous fatty acids, which indicated the stimulation of chain elongation during the synthesis of branched-chain fatty acids. The reduction of the Ri_ai-15/ai-17_ was related to the presence of exogenous fatty acids but not to the nature of these fatty acids. Although the shift to longer branched-chain fatty acids should increase the membrane melting temperature, we found WAMT values primarily affected by the melting temperatures of exogenously supplied fatty acids. WAMT values increased in all strains after supplementation with P60 due to the presence of C_16:0_ and C_18:0_ (Table 1). In addition, we detected significant differences for WAMT and MK-7 content between tested strains supplemented with P60 and P80. For *L*. *monocytogenes* strains DSM 20600^T^ and FFH, a reduced MK-7 content was detected when C_18:1_ *cis* 9 was incorporated and WAMT decreased. In contrast, strain FFL 1, which was previously reported not to have an MK-mediated temperature adaptation, increased MK-7 content in the presence of C_16:0_ and C_18:0_. These results support the previous evidence that fatty acids are not selectively incorporated as WAMT increased after supplementation with P60 compared to the non-supplemented cultures. Furthermore, our data demonstrate that the previously described MK-mediated adaptation of membrane fluidity^26^ and cell fitness^31^ in *L*. *monocytogenes* is affected by low growth temperatures and the presence of exogenous fatty acids. Thus, the interlocking of MK-mediated adaptation and FA-dependent cold adaptation is more complex as expected, as exogenous fatty acids can impact the MK content and, therefore, are also involved in the cold adaptation of *L*. *monocytogenes*.

A critical feature of this study was to confirm the incorporation of exogenous fatty acids into membrane lipids. *L*. *monocytogenes* cannot synthesize unsaturated fatty acids^27–30^. Therefore, C_18:1_ covalently linked to phospholipids PG and LPG as revealed by total Q-TOF MS must be exogenous in origin (Fig. 1a,b). All phospholipids analyzed contained one C_15:0_ acyl chain combined with a second acyl chain (C_15:0_, C_18:1_, C_16:0,_ or C_17:0_). PG and LPG species containing C_15:0_/C_15:0_ or C_15:0_/C_18:1_ were the dominating molecular species (Fig. 1c–f). Two different acyltransferases are involved in the synthesis of phospholipids in bacteria. A glycerolphosphate acyltransferase synthesizes lysophosphatidic acid, and a lysophosphatidic acid acyltransferase produces phosphatidic acid, the precursor of all phospholipids^17^. Due to the presence of C_15:0_ in all phospholipids of *L*. *monocytogenes,* one of the two acyltransferases may be characterized by a high substrate specificity for C_15:0_. In contrast, the other acyltransferase may show a broader substrate specificity for different fatty acids. Furthermore, a high similarity between the molecular species distribution regarding PG and LPG could be observed. Besides, we could detect neither C_18:1_ nor C_18:0_ in glycolipids, which are present in *L*. *monocytogenes* in addition to phospholipids^33^. Therefore, all these observations favor the enzymatic and probably selective (concerning the *sn*-position of the glycerol) incorporation of high amounts of C_18:1_ and conclusively of other exogenous fatty acids into phospholipids. These results suggest that other detected exogenous fatty acids of the fatty acid profile are also covalently bound to the polar lipids of *L*. *monocytogenes*, contrasting a previous report, which found no integration of C_18:1_ into phospholipids only the intercalation of this lipid in the bacterial membrane^34^. This discrepancy may be attributed to the use of ester-bound fatty acids (P60, P80, food extracts) in our study, in contrast to free fatty acids as a supplement in previous studies. We did not study the polar lipids in more detail, as the polar head groups of the membrane lipids have only minor effects on the thermal membrane properties and show no changes in their composition in *L*. *monocytogenes* at low growth temperatures^35–37^.

Fatty acids with a high *T*_*m*_ (saturated, straight-chain fatty acids) decrease membrane fluidity, whereas fatty acids with a low *T*_*m*_ (unsaturated and branched-chain fatty acids) increase membrane fluidity^38^. TMA-DPH-dependent anisotropy measurements confirmed the influence of exogenous fatty acids on the membrane fluidity of whole living cells with a complex lipid composition (Fig. 2). None of the strains showed the two typical plateaus that indicate the two temperature-dependent ultimate states of biomembranes: the gel-like solid-state (high TMA-DPH anisotropy) and the liquid-crystalline liquid-state (low TMA-DPH anisotropy). A linear curve progression rather than a sigmoidal curve described the relation between anisotropy and measuring temperature for all strains tested, which generally describes the phase transition of the membrane^39^. The cultures grown in TSB-YE supplemented with P80 or SSE showed a higher membrane fluidity in all strains than those without supplementation. These results indicated significantly more fluid membrane below 20 °C and unchanged fluidity above 20 °C. The change in TMA DPH anisotropy with a value of 0.03 was approximately the same for all three strains. After supplementation with P80 and SSE (Tables 1, 3), the altered fatty acid profile suggests that the exogenous unsaturated fatty acids with low *T*_*m*_ cause this effect, resulting in more beneficial membrane fluidity and more pronounced adaptation of the membrane to low temperatures. Because washed cells incubated in Ringer's solution and supplemented with P80 and SSE did neither show the implementation of supplemented fatty acids nor any change in TMA DPH anisotropy, we concluded that active incorporation of exogenous fatty acids in growing cells is a prerequisite for impacting cell membrane fluidity by these exogenous lipids (Fig. 3).

We also demonstrated that cell membranes complemented with exogenous and low *T*_*m*_ fatty acids, such as C_18:1_ *cis* 9, are protective against freeze-thaw stress. Resistance against freeze-thaw stress is used to indicate a resilient and robust membrane structure^40^. Significantly higher resistance was detected as log_10_-reduction of CFU mL^−1^ for all strains grown at 6 °C with incorporated C_18:1_ *cis* 9. In contrast, only strain DSM 20600^T^ showed lower log_10_-reduction of CFU mL^−1^ after C_16:0_ and C_18:0_ incorporation. The strains FFH and FFL 1 showed no significant changes in the log_10_-reduction of CFU mL^−1^ when C_16:0_ was incorporated. Furthermore, bacterial cell growth was reduced after incorporation of C_16:0_ and C_18:0_ and increased after the incorporation of C_18:1_ in all strains grown at 6 °C. On the other hand, if cells were grown at 37 °C, both fatty acids increased the growth rate for all strains (Fig. 5).

Yao et al.^17^ stated that *L*. *monocytogenes* do not actively incorporate exogenous fatty acids into their membrane phospholipids. Nevertheless, the genome encodes the gene loci *lmo1814*, *lmo1863,* and *lmo2514*, representing homologs of the two-component fatty acid kinase system FakA/FakB of *S*. *aureus*. For *S. aureus,* this system catalyzes the first steps in exogenous fatty acid incorporation, which is the binding and phosphorylation of exogenous fatty acids. The acyl-phosphates formed can then enter the phospholipid synthesis^20,41^. A standard nucleotide Basic Local Alignment Search Tool (BLAST) check revealed that homologs of the FakA/FakB genes are present in at least 100 deposited genomes of *L*. *monocytogenes*, highlighting the conservation of these genes and the crucial importance of this mechanism. Furthermore, we confirmed the presence of *lmo1814*, *lmo1863*, and *lmo2514* in all strains used in this study by specific PCR and subsequent sequencing analysis of the PCR products.

In this study, we could demonstrate that the fatty acid profile of *L*. *monocytogenes* was modified by exogenous fatty acids at low and high growth temperatures, changing membrane fluidity and growth properties. The present exogenous fatty acid improves membrane fluidity and cell viability at low growth temperatures. The influence of external fatty acids from the food matrix significantly affects the contamination dynamics of chilled foods. We demonstrated that the acyl chain composition plays a crucial role in the survival of *L*. *monocytogenes*, and an increase in straight-chain fatty acids reduces the organism's growth rate.

## Materials and methods

### Materials

All chemical reagents and solvents were purchased from Alfa Aesar, Carl Roth, MilliporeSigma, Sigma-Aldrich, Thermo Fisher Scientific, or VWR. All solvents and water for analytics were HPLC grade and used as received. Ultra-high temperature processed milk (3.5% fat), modified atmosphere packaged minced meat, and pre-cut vacuum-packed smoked salmon were purchased at a local supermarket chain store.

### Strains, culture media, and cultivation

In this research, three different strains of *L*. *monocytogenes* were examined. Strain FFH (= DSM 112142; serovar group 4b, lineage I) was isolated from minced meat in 2011 and strain FFL 1 (= DSM 112143; serovar group 1/2a or 3a, lineage II) from smoked salmon in 2012. In addition, the strain *L. monocytogenes* DSM 20600^T^ (serovar group 1/2a, lineage II) was obtained from the Leibniz Institute DSMZ-German Collection of Microorganisms and Cell Cultures GmbH. The adaptive response of strain FFL 1 to low temperature is primarily an FA-dependent mechanism, while strains DSM 20600^T^ and FFH also expressed an MK-based response^26^.

All strains were aerobically cultured in 200 µL or 100 mL TSB-YE. The medium is composed of tryptic soy broth containing 17 g peptone from casein L^−1^, 3 g peptone from soy meal L^−1^, 2.5 g d-glucose L^−1^, 5 g sodium chloride L^−1^, and 2.5 g dipotassium hydrogen phosphate L^−1^ supplemented with 6 g yeast extract L^−1^ or in BHI broth composed of 12.5 g brain infusion solids L^−1^, 5 g beef heart infusion solids L^−1^, 10 g protease peptone L^−1^, 2 g glucose L^−1^, 5 g sodium chloride L^−1^ and 2.5 g disodium phosphate L^−1^ using 96-well microplates or 300 mL Erlenmeyer flasks, respectively. The TSB-YE was supplemented with 0.1% (wt/vol) polysorbate 60 (P60), with 0.1% (wt/vol) polysorbate 80 (P80), with each of 0.05% (wt/vol) P60 and P80 (P60P80), with 0.1% (wt/vol) ME, with 0.1% (wt/vol) MME, or with 0.1% (wt/vol) SSE, respectively. 0.078% (wt/vol) d-sorbitol was used as control. We measured the medium's *a*_*w*_ with a LabMaster-aw instrument (Novasina, Switzerland). OD_625_ documented growth in TSB-YE with or without supplementation by a GENESYS 30 visible spectrophotometer (Thermo Fisher Scientific, USA) or a Synergy H1 modular multimode microplate reader (BioTek Instruments, Inc., USA). Growth was fitted by the Gompertz growth model as previously described^42^. Cultures were prepared in multiple independent replicates, inoculated with 1% (vol/vol) of an overnight culture at 30 °C and incubated on an orbital shaker at 6 or 37 °C and 150 rpm until late exponential phase (OD_625_ = 0.8–1.0) or stationary phase for growth rate determination. Cultures were harvested by centrifugation (10 min at 10,000 × *g*) at growth temperature and washed thrice with sterile phosphate-buffered saline (PBS) pH 7.4. Subsequently, bacterial cells were used for temperature stress tests, fatty acid analysis, determination of MK content, polar lipid analysis, and membrane fluidity analysis. Colonies were cultivated on TSA-YE at 30 °C. Additionally, each strain was incubated on TSA-YE supplemented with 0.1% (wt/vol) ME, with 0.1% (wt/vol) MME, or with 0.1% (wt/vol) SSE for fatty acid analysis.

To determine colony forming units (CFU) for the freeze-thaw stress test, 50 µL of serial dilutions were plated on TSA-YE (90 mm Petri dish) using the exponential mode (ISO 4833-2, ISO 7218, and AOAC 977.27) of the easySpiral automatic plater (Interscience, France). After a one-day incubation at 37 °C, the CFU were counted for the corresponding dilution steps, and the weighted average of enumerated *L*. *monocytogenes* was given in CFU mL^−1^. The results for the temperature stress test were presented as decadic logarithm (log_10_) reduction relative to the initial CFU mL^−1^, respectively.

### Lipid extraction from food

Total lipids from commercially available milk (3.5% fat), minced meat, and smoked salmon were extracted using the method by Bligh and Dyer as previously described^43^. Minced meat and smoked salmon were used directly without any pretreatments. Milk was freeze-dried before extraction. One hundred grams of food were incubated for 2 h at room temperature under shaking with 150 mL of chloroform/methanol (1:2, vol/vol) in a 500 mL Erlenmeyer flask. Then we added 50 mL of chloroform and let the mixture shake for another 1 h. The extract was filtered using cellulose filter paper. For phase separation, 90 mL of PBS (pH 7.4) were added, mixed vigorously, and incubated at − 20 °C. The lower layer was evaporated to dryness with nitrogen and stored at − 20 °C.

### Freeze-thaw stress tests

The freeze-thaw stress test was performed by subjecting each strain to three freeze-thaw cycles. First, three aliquots of 2 mL bacterial cell suspension for each strain were frozen at − 20 °C. Then, after 24, 48, and 72 h, aliquots were thawed for 20 min at room temperature, and the number of CFU mL^−1^ was determined. Finally, the remaining aliquots were refrozen for subsequent freeze-thaw cycles.

### Fatty acid extraction and analysis

Approximately 40 mg of washed bacterial cells per sample were used for fatty acid analysis. Fatty acids were extracted and analyzed as methyl esters (FAMEs) as previously described^26^. First, cells were resuspended in 1 mL of 15% (wt/vol) sodium hydroxide (NaOH) in methanol/water (1:1, vol/vol) using 10 mL hydrolysis tubes and saponified for 30 min at 100 °C. Next, fatty acids were methylated with 2 mL (6 N) hydrochloric acid/methanol (1:1.2, vol/vol) for 10 min at 80 °C and immediately cooled on ice. Next, fatty acid methyl esters were extracted with 1.25 mL hexane/methyl *tert*-butyl ether (1:1, vol/vol) for 10 min in an overhead mixer. Phases were separated by centrifugation (5 min at 3000 × *g*), and the lower phase was discarded. Subsequently, a base wash of the upper phase was performed with 3 mL of 1.2% (wt/vol) NaOH in water. The fatty acid methyl esters were identified by gas chromatography-mass spectrometry (GC-–MS) with a 7890A gas chromatograph (Agilent Technologies, USA) equipped with a 5% phenylmethyl silicone capillary column coupled with a 5975C mass spectrometer (Agilent Technologies, USA), as previously described^44^. Fatty acid analysis was performed with MSD ChemStation software (version E.02.00.493, Agilent Technologies, USA), and their retention times and mass spectra were identified. In addition, dimethyl disulfide (DMDS) derivatization and analyses of unsaturated FAMEs were performed as described by Nichols et al.^45^.

The effect of alterations in fatty acid profiles associated with the supplemented lipids on membrane fluidity was determined by calculating the weighted average melting temperature (WAMT) as described previously^26^. Considering the individual melting temperatures of each FA, this parameter allows integrating the quantitative changes of all membrane-associated fatty acids. However, the WAMT value does not represent the actual melting temperature of the cytoplasmic membrane, which also depends on the total polar lipid structure. Therefore, the melting temperatures for fatty acids were taken from previously described research^46,47^.

1 $$\mathrm{WAMT}={\mathrm{FA}}_{1}\,(\%)\times {\mathrm{T}}_{\mathrm{m}}\left({\mathrm{FA}}_{1}\right)+{\mathrm{FA}}_{2}\left(\mathrm{\%}\right)\times {\mathrm{T}}_{\mathrm{m}}\left({\mathrm{FA}}_{2}\right)+ \dots + {\mathrm{FA}}_{\mathrm{n}}\left(\mathrm{\%}\right)\times {\mathrm{T}}_{\mathrm{m}}\left({\mathrm{FA}}_{\mathrm{n}}\right)$$

The bacterial membrane's weighted average melting temperature (WAMT) was calculated according to equation 1. All fatty acids (FA_1_ to FA_n_) that are present in the fatty acid profile, FA_1_ (%) is the percentage of fatty acid no. 1 and melting temperature (*T*_*m*_) of the corresponding fatty acid. The difference in WAMT (ΔWAMT) indicates the extent of adaptation through the fatty acid shift.

### Polar lipid extraction and analysis

The mass spectra of polar lipids were analyzed to verify whether the fatty acids from the supplemented culture media were covalently linked to polar lipids of the bacterial membrane. Total lipids from bacterial cells were extracted according to Bligh and Dyer^48^. Approximately 50 mg bacterial cells were resuspended in 3 mL H_2_O and boiled for 10 min using 10 mL hydrolysis tubes. Ruptured cells were centrifuged (15 min at 3000 × *g*), and the supernatant was discarded. The extraction was performed in two steps using 3 mL chloroform/methanol (1:2, vol/vol) and 3 mL chloroform/methanol (2:1 vol/vol) under shaking for 30 min. Extracts were pooled, and phases were separated by adding 3 mL chloroform and 0.75 mL water (with a final ratio of chloroform/methanol/water of 2:1:0.75, vol/vol/vol) followed by centrifugation (15 min at 3000 × *g*). The organic phase was evaporated to dryness with nitrogen and stored at − 80 °C until analysis. For analysis, the evaporated extracts were dissolved in 0.1 mL with chloroform/methanol (2:1 vol/vol) and filtered through 0.2 μm polytetrafluoroethylene (PTFE) filters (VWR International, Germany).

Membrane lipids were analyzed using a 6530 Q-TOF MS (Agilent Technologies, United States) by direct infusion of total lipid extracts in the positive ion mode^49^. PG and LPG were additionally measured in the negative ion mode with 50 V collision energy. Lipids were selected in a non-targeted approach in the “auto-MS/MS” mode, which means that the most intense precursor ions are selected automatically. Glycolipids were separated by solid-phase extraction of total lipid extracts with Isolute SI Columns (Biotage AB, Sweden) before analyzing with Q-TOF MS. The data acquisition was performed with MassHunter software (version B.02.00; Agilent Technologies, USA).

### Isoprenoid quinone extraction and analysis

About 30–50 mg cells were extracted with methanol/chloroform (9:5, vol/vol) as previously described^26,50^. Evaporated extracts were made up to 1 mL with methanol and analyzed using a 1260 Infinity Quaternary LC system (Agilent Technologies, USA) equipped with a quaternary pump, an autosampler, a thermo-controlled column compartment, and a diode array detector. Compounds were separated isocratically at 30 °C on a Hypersil™ ODS C18 column (Thermo Fisher Scientific, USA) using methanol/diisopropyl ether (9:2, vol/vol) as eluent (flow rate of 1 mL min^−1^). Isoprenoid quinones were detected at 270 and 275 nm and were identified by their absorption spectrum and retention time. The quinones were quantified as vitamin K_1_ equivalents using an external calibration curve and an internal vitamin K_1_ standard. Data acquisition was performed with OpenLAB CDS ChemStation software (version C.01.07, Agilent Technologies, USA).

### Membrane fluidity analyses by anisotropy

Whole bacterial cells were prepared and stained with the fluorescent probe TMA-DPH to determine anisotropy, as Seel et al.^26^ described. TMA-DPH anisotropy is particularly suitable for measuring membrane fluidity measuring the direct mobility of the probe and adjacent lipids^39^. High TMA-DPH anisotropy values correspond to low fluidity and low values to high membrane fluidity. Steady-state fluorescence was measured in an LS 55 fluorescence spectrometer combined with a PTP-1 Peltier system (PerkinElmer, United States) for sample temperature regulation. Cells were washed and resuspended in Tris-EDTA (TE) buffer solution (pH 7.4) and diluted to OD_625_ 0.2. TMA-DPH stock solution was prepared in dimethyl sulfoxide (DMSO) at a concentration of 0.4 mM. Cells were stained with 0.5 µM TMA-DPH for 10 min at 30 °C in the dark and washed twice. Measurements were performed with a 2 mL sample volume in 3.5 mL quartz glass cuvettes (Hellma, Germany). For TMA-DPH anisotropy measurements, samples were excited at 355 nm, and emission intensities were recorded at 425 nm. Anisotropy (*r*) values were calculated from polarized intensities using Eq. (2).

2 $$r = \frac{{I}_{\mathrm{VV}}-G{I}_{\mathrm{VH}}}{{I}_{\mathrm{VV}}+2G{I}_{\mathrm{VH}}}$$

Anisotropy (*r*) of trimethylammonium diphenylhexatriene (TMA-DPH) was calculated according to equation 2. The fluorescence intensity (*I*) from which blank values of non-dyed cells were subtracted. Grating factor (*G*), calculated by the ratio of horizontal (H) and vertical (V) polarizer positions for the excited and the emitted light. Each data point was calculated from 10–20 single measurements.

The abiotic effect of P80 on biomembranes was tested with non-growing *L*. *monocytogenes* cells. For this purpose, bacterial cells were grown in TSB, washed thrice (10 min at 3000 × *g*) with Tris–EDTA (TE) buffer (Alfa Aesar, USA) solution (pH 7.4). The bacterial cell pellet was then resuspended in 100 mL TE buffer to OD_625nm_ 0.8 without or with 0.1% (wt/vol) P80 and incubated at 6 °C for 24, 48, and 78 h. After each time point, the membrane fluidity, OD_625_, and fatty acid profile were determined as described previously.

### Statistical analysis

Statistical analysis was performed using Prism (version 9.2.0; GraphPad Software, USA). Mean values (M) and standard deviations (SD) of *n* (see legends) biological replicates were calculated for all experiments. Two-way ANOVA was performed with recommended post hoc test (α = 0.001). Data are presented as M ± SD from separate experiments; *p < 0.001, **p < 0.0001, ***p < 0.00001, ****p < 0.000001.
